# High-density thrombus and maximum transverse diameter on multi-spiral computed tomography angiography combine to predict abdominal aortic aneurysm rupture

**DOI:** 10.3389/fcvm.2022.951264

**Published:** 2022-09-30

**Authors:** Heqian Liu, Zhipeng Chen, Chen Tang, Haijian Fan, Xiaoli Mai, Jing Cai, Tong Qiao

**Affiliations:** ^1^Department of Vascular Surgery, Nanjing Drum Tower Hospital Clinical College of Xuzhou Medical University, Nanjing, China; ^2^Department of Vascular Surgery, Nanjing Drum Tower Hospital, The Affiliated Hospital of Nanjing University Medical School, Nanjing, China; ^3^Department of Radiology, Nanjing Drum Tower Hospital, The Affiliated Hospital of Nanjing University Medical School, Nanjing, China

**Keywords:** ruptured abdominal aortic aneurysm, intraluminal thrombus, high-density, MSCTA, maximum transverse diameter

## Abstract

**Objective:**

We attempted to measure maximum transverse diameter (MTD) of and CT values of ILT by using multi-spiral computed tomography angiography (MSCTA) to investigate the predictive value of MTD with different CT values of thrombus on the risk of AAA rupture.

**Methods:**

Forty-five intact abdominal aortic aneurysms (IAAA) and 17 ruptured abdominal aortic aneurysms (RAAA) were included in this study. MTD and CT values in their planes were measured from MSCTA images and aneurysm lumen and thrombus volumes were calculated for the range of different CT values.

**Results:**

The median of maximum CT value of thrombus at the plane of MTD was higher in RAAA (107.0 HU) than the median in IAAA (84.5 HU) (*P* < 0.001). Univariate logistic regression analysis showed that the maximum CT value was a risk factor for RAAA (*P* < 0.001). It was further found that the area under the ROC curve for thrombus maximum CT value in the MTD plane to predict RAAA was 0.848 (*P* < 0.001), with a cut-off value of 97.5 HU, a sensitivity of 82.35%, and a specificity of 84.44%. And the MTD of the abnormal lumen combined with the maximum CT value at its plane predicted RAAA with an area under the ROC curve of 0.901, a sensitivity of 76.47%, and a specificity of 97.78%. The further analysis of thrombus volume in the range of different CT value showed that median thrombus volume in RAAA in the range of 30 HU~150 HU was 124.2 cm^3^ which was higher than the median of 81.4 cm^3^ in IAAA (*P* = 0.005). To exclude confounding factors (aneurysm volume), we calculated the standardized thrombus (ILT volume/total aneurysm volume), and the thrombus volume in the range of 30 HU~150 HU in RAAA was positively correlated with the standardized thrombus volume (ρ = 0.885, *P* < 0.001), while the thrombus volume in the range of −100 HU~30 HU was not correlated with it (ρ = 0.309, *P* = 0.228).

**Conclusions:**

High-density ILT shown on MSCTA in AAAs is associated with aneurysm rupture, and its maximum transverse diameter combined with the maximum CT value in its plane is a better predictor of RAAA.

## Introduction

Rupture is one of the most serious complications of abdominal aortic aneurysm (AAA), and its morbidity and mortality rates are extremely high. It has been reported in the literature that 59–83% of patients with ruptured abdominal aortic aneurysm (RAAA) die before reaching the hospital, and even after timely arrival and medical intervention, the morbidity and mortality rate are as high as 30–80% (1). Therefore, the factors that cause abdominal aortic rupture should be taken into account. There are several factors that can cause AAA rupture in previous studies, such as increased diameter or volume (2), bacterial or fungal infection (3), etc. In the present day, national and international research institutions basically agree that the risk of AAA rupture is assessed mainly by the maximum transverse diameter (MTD) of the abdominal aorta (4, 5). Although the MTD has been shown to predict rupture in population studies such as the UK Small Aneurysm Trial (6) and the Aneurysm Detection and Management Trial (7), it provides a poor assessment of individual risk while some large-diameter AAAs have been found to remain stable over a lifetime (8). There are some controversies as to whether the aneurysm diameter alone can be a reason for intervention (9). Therefore, investigating specific rupture factors will probably help to reduce the risk of aneurysm rupture.

Intraluminal thrombus (ILT) is one of the common substances in AAA. ILT has been suggested in previous studies to reduce mural stress in AAA and thus may prevent rupture of AAA (10–12). In contrast, some other studies has found that ILT can act as an inflammatory lesion of protein hydrolysis and aortic wall degeneration, thus increasing the risk of AAA rupture (13–15). While the above studies only address the effect of ILT in AAA as a whole, some studies in recent years have already started to focus on the relationship between different Hounsfield Units (HU) within the tissue and disease under computed tomography angiography (CTA). For example, some studies has reported correlation between the density of coronary arteries (16) and carotid plaques (17) on computed tomography and the stability of the atherosclerotic lesions. Banno et al. (18) analyzed the correlation between thrombus and spinal cord ischemia based on thrombus CTA density in the range of different CT value in a thoracic aortic aneurysm study. However, it has not been reported whether there is a differential effect of thrombus on RAAA in the range of different CT value. Our study rely primarily on aortic MSCTA to assess the potential association of MTD and different CT values of thrombus with RAAA.

## Data and methods

### Research subjects

**Figure 1:**
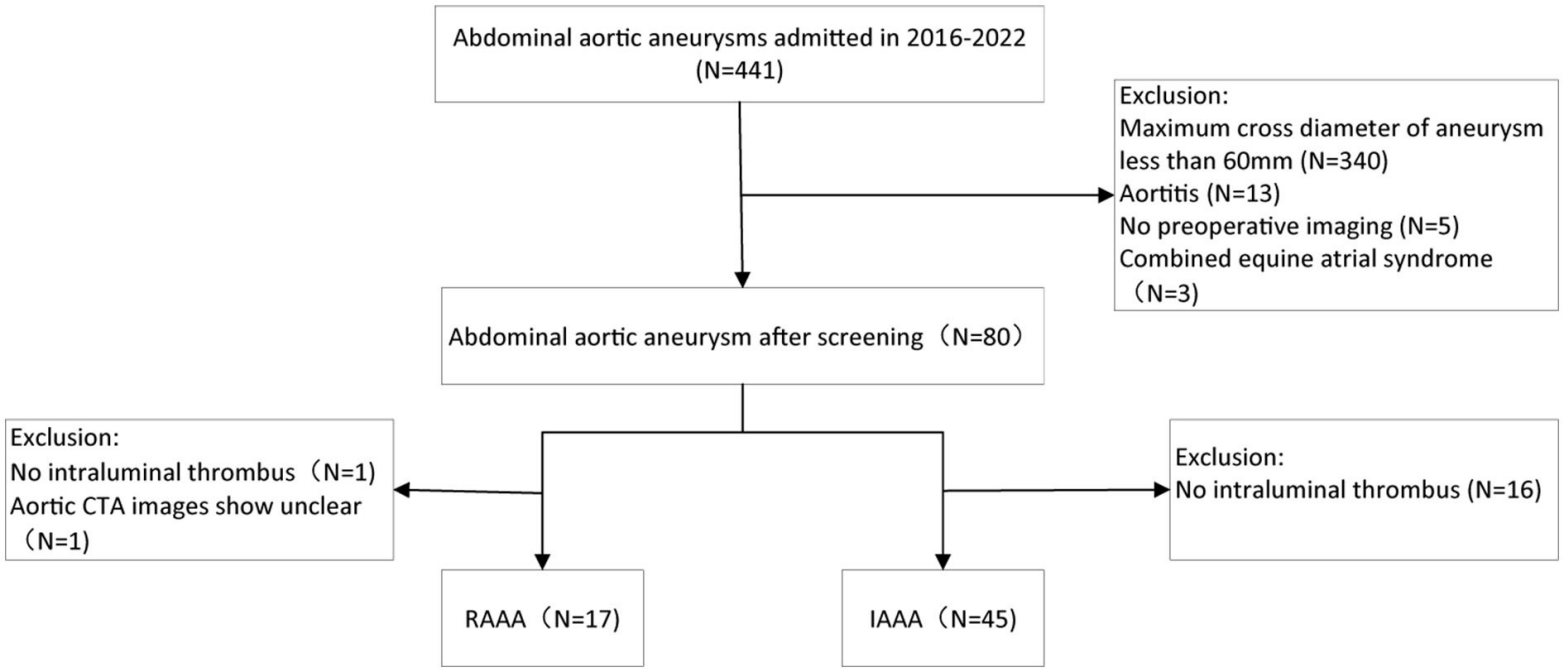
Flow chart of the patient selection.

Patients with intact abdominal aortic aneurysm (IAAA) and RAAA who attended Hospital from January 2016 to January 2022 were included and divided into two groups on this basis. Inclusion criteria: (1) patients with IAAA or RAAA clearly diagnosed through CTA (diameter ≥60 mm); (2) complete imaging data. Exclusion criteria: (1) no images of CTA of the abdominal aorta; (2) no ILT in the abdominal aorta; (3) Aortitis; (4) infected abdominal aortic aneurysm; (5) stable syndrome and other genetic diseases. Therefore, the clinical data of a total of 62 patients were finally analyzed in this study. Signing informed consent was exempted because the patients' identities were anonymous, and this retrospective study was approved by the Ethics Committee of Nanjing Drum Tower Hospital (Figure 1).

## Research methods

### Data collection

**Figure 2:**
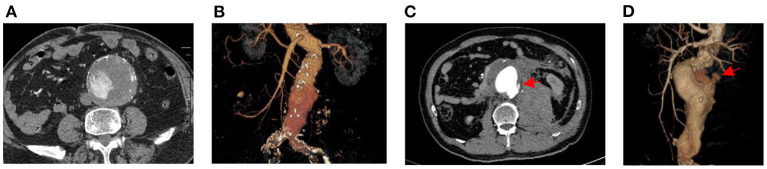
MSCTA images of intact and ruptured abdominal aortic aneurysms. **(A)** shows the cross-section image of the intact abdominal aortic aneurysm. **(B)** shows the three-dimensional image of the intact abdominal aortic aneurysm. **(C)** shows the cross-section image of the ruptured abdominal aortic aneurysm and **(D)** shows the three-dimensional image of the ruptured abdominal aortic aneurysm. The red arrow indicates the location of the ruptured wall of the abdominal aortic aneurysm.

This study was a cross-sectional study and patients were screened according to inclusion and exclusion criteria. Their previous underlying medical history, clinical base data, and imaging data were collected from the electronic medical record system. The history of previous underlying diseases included hypertension, diabetes mellitus, cerebral infarction and coronary heart disease; clinical base information included gender, age, BMI, history of smoking and alcohol consumption; and imaging data mainly consisted of abdominal aortic MSCTA (Figure 2).

### CTA analysis of enrolled patients

**Figure 3:**
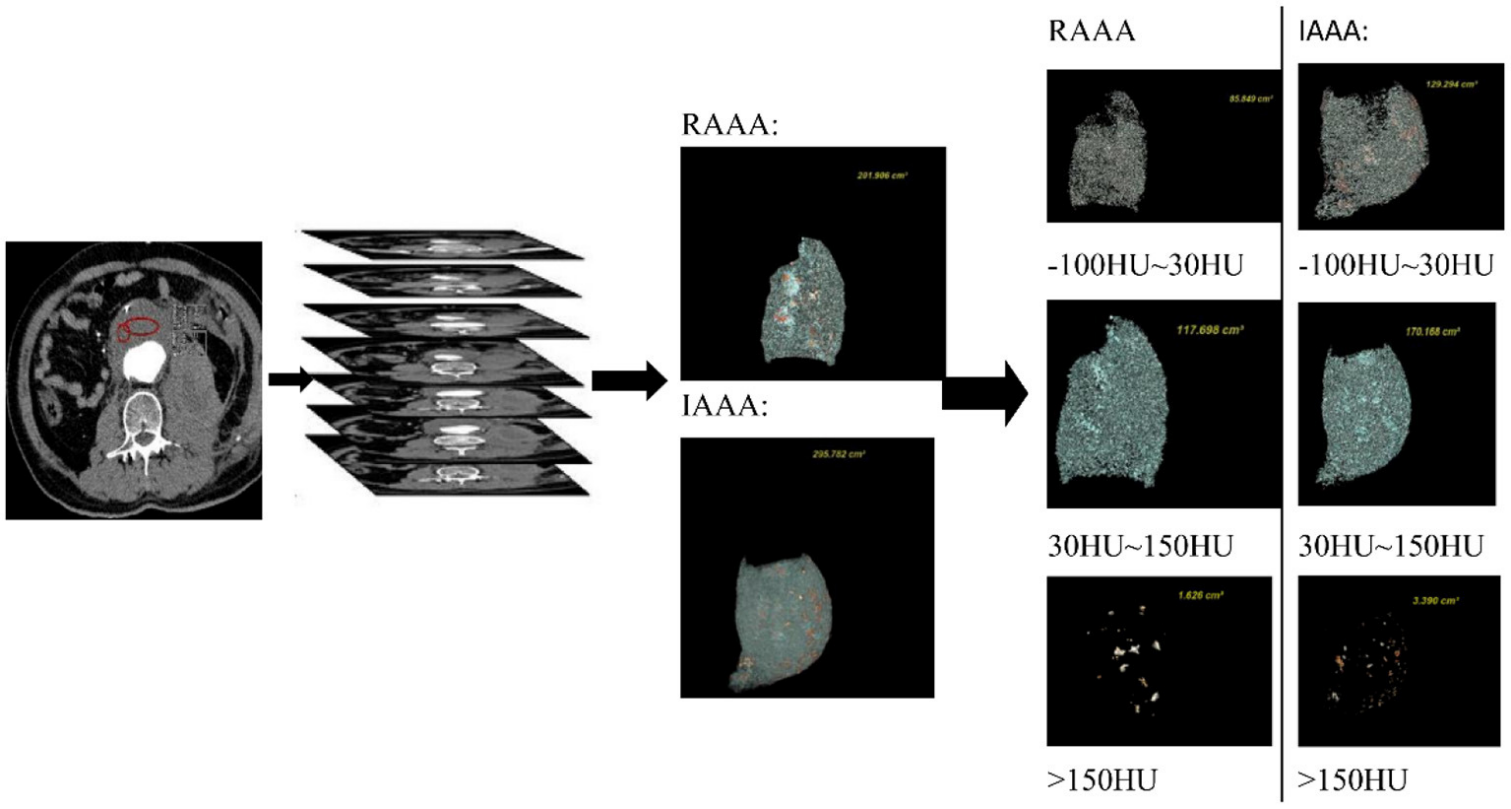
The procedure of measuring CT values at the maximum transverse diameter and calculating thrombus volumes in the range of different CT in IAAA and RAAA.

All MSCTA analyses were performed by two professional imaging physicians, and the patient's clinical symptoms and characteristics were not reviewed. The MSCTA analysis was performed in four different phases, the first of which was to assess the quality of the images included in the patient and to exclude any abdominal aorta that showed artifacts or degrading motion artifacts in the image quality; The second phase was to find the largest transverse cross-sectional image and two imaging physicians measured the minimum CT, average CT and maximum CT of ILT in this plane by using AW443 workstation, and checked the consistency of repeated measurements. We judged the intra-group correlation coefficient of the reproducibility evaluation of the measurements based on the estimation of Single Measures, i.e., ICC = 0.811 (*P* < 0.001). Therefore, the reproducibility of CT values tested by two imaging physicians in this study was good. The final results were obtained by averaging the values measured by the two imaging physicians. The third stage is to reconstruct the AAA in 3D using the AW443 imaging workstation to outline the region of interest (ROI) of the abdominal aorta in thin layer CT images (the precise scope of the AAA is from the infrarenal abdominal aorta to above the common iliac artery bifurcation). The thrombus was reconstructed again using the same outline and its volume was calculated; In the four stage, the corresponding volumes were obtained by entering different ranges of CT values in the AW443 workstation. The classification in this study concerning the different CT values in the range is based on the most commonly used classification in the field of coronary arteries (19): (−100 HU~30 HU), (30 HU~150 HU) 和(>150 HU) (Figure 3).

### Thrombus thickness and percentage

The thrombus thickness is measured in the plane of the largest transverse diameter of the aneurysm, starting at the edge of the aneurysm wall and ending at the thickest point of the thrombus. Percentage of thrombus thickness = thrombus thickness/maximum aneurysm diameter.

### Standardized thrombus volume

ILT and intraluminal volumes were calculated using CTA through the AW443 workstation, and the percentage of total aneurysm volume accounted for the ILT was calculated for each patient: Standardized thrombus volume = ILT volume/ total AAA intraluminal volume.

### Statistical analysis

Data were analyzed using the statistical software R (http://www.R-project.org, The R Foundation) and SPSS 28.0. Measured data conforming the normal distribution were expressed as $\bar{X}$ ± s, and the *t*-test was used for comparison of means between groups. Bias distributed variables were expressed as M (Q1, Q3), and the Mann-Whitney *U* test was used for comparison between groups. Qualitative data were expressed as rates or composition ratios, and comparisons between groups were made using the χ^2^ test. The Receiver Operating Characteristic (ROC) curve was used to assess the sensitivity and specificity of clinical variables in predicting RAAA and to calculate cutoff values. Univariate logistic regression analysis was used to examine the association between RAAA and clinical variables. Quantitative skewed distribution variable correlation analysis was performed by using spearman, and the correlation coefficient was ρ. *P* < 0.05 was considered to be statistically significant.

The sample size was calculated by using PASS software for *post-hoc* efficacy analysis, and the maximum CT value had sufficient efficacy to detect differences between the two groups at an a error of 0.05 (efficacy = 0.99947).

## Results

### Baseline information of the study populations

A total of 441 patients with abdominal aortic aneurysm were admitted to our hospital. Among the collected patients with RAAAs, only one had a MTD of <60 mm in the abdominal aorta and one had no intraluminal thrombus, thus two patients were excluded. And one more was excluded because of unclear CTA images, thus causing a large difference in CT number measured by the two imagers, and was excluded after final discussion. To prevent errors due to large differences in the MTD, we analogized the lowest diameter of the collected RAAA and selected intact AAA larger than 60 mm. And we excluded non-ILT patients as there was only one case of non-ILT in the RAAA from our data. Therefore, in order to control for confounding factors, patients without ILT in the IAAA were also excluded. Finally, A total of 62 patients with AAA were included in this study, of which 45 were in the IAAA group and 17 in the RAAA group. There were 41 (91.1%) males and 4 (8.9%) females among the 45 IAAA patients, and their age was 75.1 ± 7.1 years. This compares to the age of 69.7 ± 6.9 years in the 17 RAAA patients, 13 (76.5%) of whom were male subjects. Baseline characteristics of all study populations are summarized in Table 1. Overall, RAAA patients were younger than IAAA (*P* = 0.008), while the weight of 23.9 ± 2.9 Kg/m^2^ in the IAAA group was not significantly different from 24.2 ± 2.5 Kg/m^2^ in the IAAA group, and the discrepancy between the two groups was not statistically significant (*P* = 0.796). There was also no significant difference in the history of male, hypertension, diabetes mellitus, cerebral infarction and coronary artery disease between the two groups (all *P* > 0.05). In blood pressure testing at admission, we found higher systolic (*P* < 0.001) and diastolic blood pressure (*P* = 0.001) in the IAAA group compared to the RAAA group.

**Table 1:** Baseline information of intact and ruptured abdominal aortic aneurysms.

**Variables**	**Total (*n* = 62)**	**IAAA group (*n* = 45)**	**RAAA group (n = 17)**	***P*** **-value**
Sex, *n* (%)				0.198
Female	8 (12.9)	4 (8.9)	4 (23.5)	
Male	54 (87.1)	41 (91.1)	13 (76.5)	
Age (years)	72.0 (69.0, 77.8)	74.0 (70.0, 81.0)	69.0 (65.0, 72.0)	0.008
BMI (Kg/m^2^)	24.0 ± 2.8	23.9 ± 2.9	24.2 ± 2.5	0.796
Hypertension, *n* (%)				0.164
No	14 (23.0)	8 (17.8)	6 (37.5)	
Yes	47 (77.0)	37 (82.2)	10 (62.5)	
Diabetes, *n* (%)				1.000
No	49 (80.3)	36 (80)	13 (81.2)	
Yes	12 (19.7)	9 (20)	3 (18.8)	
CI, *n* (%)				0.259
No	50 (82.0)	35 (77.8)	15 (93.8)	
Yes	11 (18.0)	10 (22.2)	1 (6.2)	
CHD, *n* (%)				0.174
No	54 (88.5)	38 (84.4)	16 (100)	
Yes	7 (11.5)	7 (15.6)	0 (0)	
SBP (mm/Hg)	124.8 ± 23.7	131.0 ± 17.5	101.3 ± 29.5	<0.001
DBP (mm/Hg)	73.8 ± 14.4	77.0 ± 11.4	61.7 ± 18.1	0.001

IAAA, Intact abdominal aortic aneurysm; RAAA, Ruptured abdominal aortic aneurysm; SBP, Systolic blood pressure; DBP, Diastolic blood pressure; CI, Cerebral infarction; CHD, Coronary heart disease.

### ILT in the study populations

Table 2 indicates the ILT in the RAAA group vs. the IAAA group. The results showed that the median 78 cm of the RAAA group had a larger MTD in the lumen compared to the median 78 cm of the IAAA group (*P* = 0.003). Further measurement of thrombus CT values at the plane of MTD showed that the difference between the minimum CT value of thrombus in the RAAA group (−15.8 ± 16.5) HU and the minimum CT value in the IAAA group (−10.5 ± 14.9) HU was not significant, and the difference between the two groups was not statistically significant (*P* = 0.231); however, the average CT value of thrombus at the plane of MTD in the RAAA group (42.9 ± 7.6) HU was higher than the average CT value of thrombus at the plane of MTD in the IAAA group (37.6 ± 5.7) HU, and the difference between the two groups was statistically significant (*P* = 0.004), and the median of the maximum CT value for thrombus at the plane of MTD in the RAAA group was 107 HU higher than the median of the maximum CT value for thrombus at the plane of the MTD in the IAAA group 84.5 HU (*P* < 0.001). We further observed the CT values in both groups in different diameters which showed that the maximum CT values measured at the plane of MTD of the abdominal aortic aneurysm were higher in the RAAA group than in the IAAA group in the diameter ranges of 60~69 mm, 70~79 mm and 80~102 mm. This suggests that the high-density thrombus shown by MSCTA in different diameter ranges has an important effect on abdominal aortic rupture (Table 3).

**Table 2:** Aneurysm lumen and thrombus in intact and ruptured abdominal aortic aneurysms.

**Variables**	**Total (*n* = 62)**	**IAAA (*n* = 45)**	**RAAA (*n* = 17)**	***P*** **-value**
Maximum transverse diameter (mm)	69.5 (65.8, 78.2)	68.0 (65.1, 74.0)	78.0 (71.9, 85.0)	0.003
Thrombus thickness	32.9 ± 11.2	32.0 ± 10.5	35.2 ± 13.0	0.33
Percentage of thrombus	46.4 ± 12.4	46.3 ± 12.5	46.7 ± 12.4	0.897
Minimum CT value (HU)	−11.5 ± 16.1	−10.5 ± 14.9	−15.8 ± 16.5	0.231
Average CT value (HU)	39.4 ± 7.3	37.6 ± 5.7	42.9 ± 7.6	0.004
Maximum CT value (HU)	85.8 (78.8, 104.5)	84.5 (78.5, 89.0)	107.0 (99.0, 115.5)	<0.001
Abdominal aortic aneurysm volume	307.1 (278.3, 392.0)	293.6 (256.2, 355.2)	392.2 (307.1, 518.3)	0.002
Total volume of thrombus in lumen	148.2 (106.1, 191.6)	128.4 (97.0, 172.2)	176.4 (153.4, 261.3)	0.004
The volume of (−100 HU~30 HU)	47.3 (27.5, 63.7)	42.2 (25.2, 60.2)	62.3 (47.2, 72.8)	0.076
The volume of (30 HU~150 HU)	96.5 (72.1, 134.8)	81.4 (71.2, 115.2)	124.2 (99.2, 197.3)	0.005
The volume of (>150 HU)	2.0 (1.0, 3.3)	2.1 (1.0, 3.4)	2.0 (0.6, 2.7)	0.825

IAAA, Intact abdominal aortic aneurysm; RAAA, Ruptured abdominal aortic aneurysm.

**Table 3:** The differences of maximum CT values at different transverse diameter ranges in the RAAA and IAAA groups.

**Variable**	**Event(n)**	**Z**	***P*** **-value**
Maximum transverse diameter			
60~69 mm	IAAA (27)	−2.95	0.003
	RAAA (4)		
70~79 mm	IAAA (12)	−2.35	0.019
	RAAA (6)		
80~102 mm	IAAA (5)	−2.03	0.042
	RAAA (7)		

IAAA, Intact abdominal aortic aneurysm; RAAA, Ruptured abdominal aortic aneurysm.

The imaging physicians outlined the target range with the AW443 workstation. In terms of the total volume within the AAA lumen, the median of 392.2 cm^3^ was higher in the RAAA group than the median of 293.6 cm^3^ in the IAAA group, and the discrepancy between the two groups was statistically significant (*P* = 0.002). The thrombus was then isolated from the lumen of the AAA, and the results showed that the median of the total thrombus volume was 176.4 cm^3^ in the RAAA group than the median of 128.4 cm^3^ in the IAAA group, and the difference between the two groups was statistically significant (*P* = 0.004). Further analysis of the thrombus volume in the range of different CT values showed that in the range of −100 HU~30 HU the thrombus volume in the RAAA group the median of thrombus volume was 62.3 cm^3^ which was not significantly correlated with the median of thrombus volume in the IAAA group that was 42.2 cm^3^, and there was no statistical difference between the two groups (*P* = 0.076); however, in the range of 30 HU~150 HU, the median thrombus volume of 124.2 cm^3^ in the RAAA group was significantly higher than the median thrombus volume of 81.4 cm^3^ in the IAAA group, and there was a statistical difference between the two groups (*P* = 0.005); The median thrombus/plaque volume of 2.0 cm^3^ in the RAAA group was not significantly different from the median thrombus/plaque volume of 2.1 cm^3^ in the IAAA group in the range of >150 HU, and there was no statistical difference between the two groups (*P* = 0.825).

### Risk factor analysis for RAAA

Table 4 shows that univariate logistic regression analysis of RAAA in relation to clinical variables. The results showed age (OR = 0.89, 95% CI = 0.81~0.98, *P* = 0.015), maximum transverse diameter (OR = 1.08, 95%CI = (1.02~1.15), *P* = 0.008), average CT value (HU) (OR = 1.14, 95%CI = 1.03~1.25), *P* = 0.009), maximum CT value (HU) (OR = 1.13, 95%CI = (1.06~1.20), *P* < 0.001) and the volume of (30 HU~150 HU) (OR = 1.01, 95% CI = (1.00~1.02), *P* = 0.023) may be strongly associated with RAAA in the univariate model.

**Table 4:** Univariate logistic regression analysis of clinical variables and RAAA.

**Variable**	**B**	**SE**	**OR (95CI%)**	***P*** **-value**
Sex (male)	−1.15	0.78	0.32 (0.69~1.45)	0.139
Age (years)	−0.12	0.05	0.89 (0.81~0.98)	0.015
BMI (Kg/m^2^)	0.04	0.13	1.04 (0.80~1.35)	0.792
Hypertension	−1.02	0.65	0.35 (0.10~1.28)	0.115
diabetes	−0.08	0.74	0.92 (0.22~3.95)	0.914
Cerebral infarction	−1.46	1.09	0.23 (0.03~1.99)	0.183
Systolic blood pressure	−0.06	0.02	0.94 (0.90~0.98)	0.002
Diastolic blood pressure	−0.08	0.03	0.92 (0.87~0.98)	0.005
Maximum transverse diameter	0.08	0.03	1.08 (1.02~1.15)	0.008
Thrombus thickness	0.03	0.03	1.03 (0.98~1.08)	0.326
Percentage of thrombus	0.003	0.03	1.00 (0.96~1.06)	0.895
CT value at the plane of maximum transverse diameter				
Minimum CT value (HU)	−0.02	0.02	0.98 (0.94~1.02)	0.230
Average CT value (HU)	0.13	0.05	1.14 (1.03~1.25)	0.009
Maximum CT value (HU)	0.12	0.03	1.13 (1.06~1.20)	<0.001
Abdominal aortic aneurysm volume	0.003	0.002	1.00 (1.00~1.01)	0.080
Total volume of thrombus in lumen	0.01	0.003	1.01 (1.00~1.02)	0.023
The volume of (−100 HU~30 HU)	0.02	0.01	1.02 (1.00~1.04)	0.071
The volume of (30 HU~150 HU)	0.01	0.01	1.01 (1.00~1.02)	0.023
The volume of (>150HU)	−0.04	0.12	0.96 (0.76~1.22)	0.753

RAAA, Ruptured abdominal aortic aneurysm.

### Maximum transverse diameter and high-density thrombus shown by MSCTA can jointly predict RAAA

**Figure 4:**
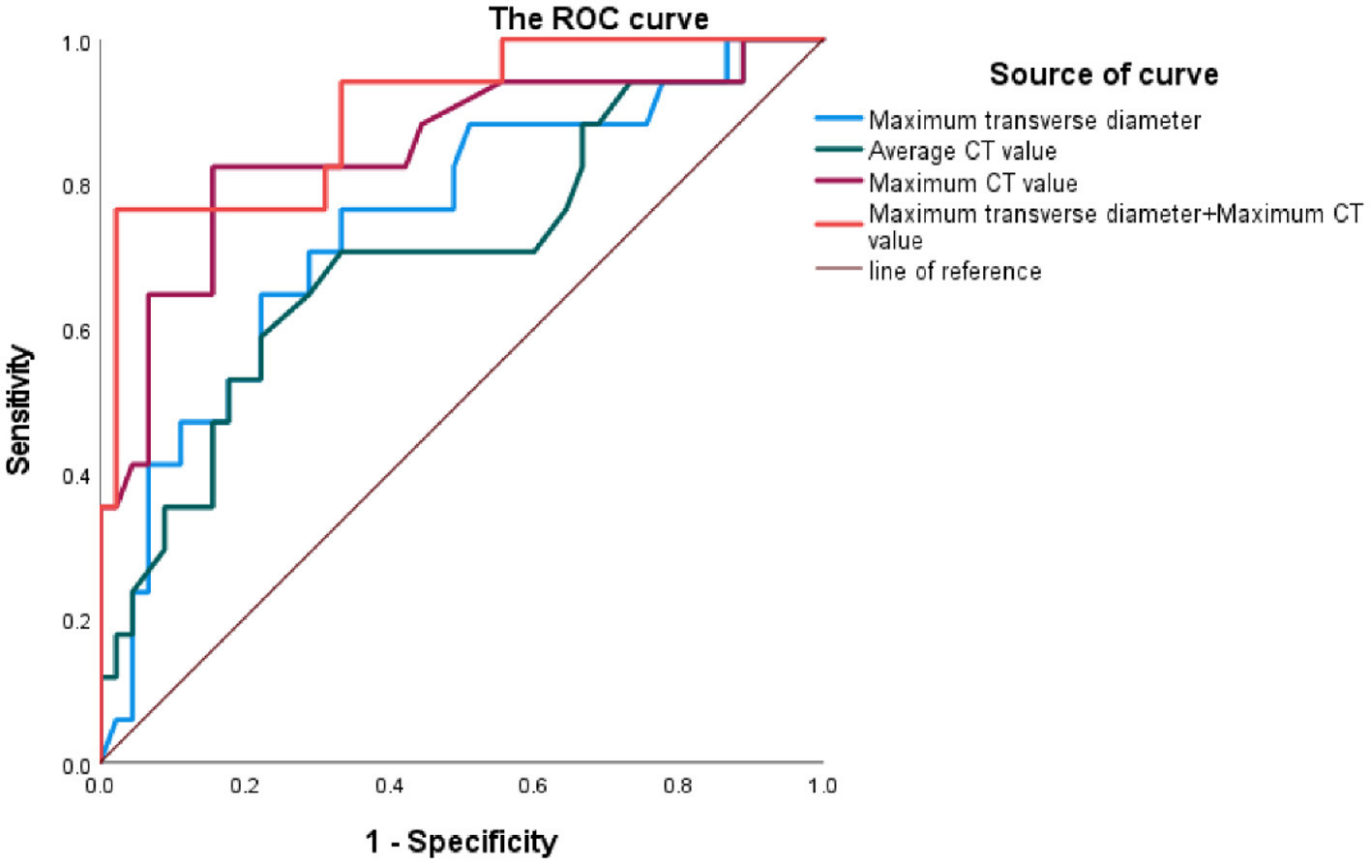
ROC curves of clinical variables and ruptured abdominal aortic aneurysm.

Figure 4 shows that the Maximum CT value measured at the plane of the MTD of the AAA lumen predicts the area under the ROC curve (AUC) for RAAA to be 0.848, with an optimal cut-off value of 97.75 HU, a sensitivity of 82.35% and a specificity of 84.44%; the average CT value measured at the plane of the MTD of the AAA lumen predicts the area under the ROC curve (AUC) for RAAA of 0.703, with the best critical value of 39.25 HU, a sensitivity of 70.59%, and a specificity of 66.67%; the MTD of the AAA lumen predicted the area under the ROC curve (AUC) for RAAA of 0.746, with the best critical value of 71.68 mm, a sensitivity of 76.47%, and a specificity of 66.67%; and the combination of AAA lumen MTD and Maximum CT value for predicting RAAA was found to have an area under the ROC curve of 0.901, a sensitivity of 76.47%, and a specificity of 97.78%. These results suggest that high-density thrombus shown by MSCTA is a good predictor for RAAA (*P* < 0.001). If MTD and Maximum CT value measured at the plane of the MTD of the AAA lumen were combined to predict RAAA, the results were better than MTD or Maximum CT value alone (*P* < 0.001) (Table 5).

**Table 5:** Clinical variables predict the area under the ROC curve for RAAA.

**Variable**	**AUC**	**Cut-off**	**Sensitivity**	**Specificity**	***P*** **-value**
Maximum CT value (HU)	0.848	97.75	82.35%	84.44%	<0.001
Average CT value (HU)	0.703	39.25	70.59%	66.67%	0.011
Maximum transverse diameter	0.746	71.68	76.47%	66.67%	0.003
Maximum transverse diameter+ Maximum CT value (HU)	0.901	-	76.47%	97.78%	<0.001

RAAA, Ruptured abdominal aortic aneurysm.

### Changes in the range of different CT value after standardized thrombosis

In order to see the trend of thrombus in the RAAA and IAAA groups in the range of different CT value, the authors converted the ILT volume in both groups to the standardized thrombus volume (ILT volume / total AAA intraluminal volume). To understand the correlation, the authors made spearman correlation analysis between thrombus volumes in the range of different CT value and standardized thrombus, and Table 6 shows that in the IAAA group CT values of thrombus in the range of 30 HU~150 HU and −100 HU~30 HU volumes were positively correlated with standardized thrombus volumes, with correlation coefficients (ρ) of 0.667 and 0.652, respectively (both *P* < 0.001). In the RAAA group CT values of thrombus in the range of 30 HU~150 HU were positively correlated with standardized thrombus volume (ρ = 0.885, *P* < 0.001), whereas CT values of thrombus in the range of −100 HU~30 HU were not correlated with standardized thrombus volume (ρ = 0.3091, *P* = 0.228).

**Table 6:** Correlation between thrombus volume in different CT value ranges and standardized thrombus.

**Thrombus volume in different CT value ranges**	**IAAA group**		**RAAA group**	
	**ρ**	***P***	**ρ**	***P***
−100 HU~30 HU	0.652	<0.001	0.309	0.228
30 HU~150 HU	0.667	<0.001	0.885	<0.001

Standardized thrombus, ILT volume/total aneurysm volume.

## Discussion

Our study collected patients with AAA (MTD ≥60 mm) from January 2016 to January 2022 in the Department of Vascular Surgery. The aim of this study was to investigate the association of high-density thrombus shown by MSCTA with RAAA. The results showed that high-density thrombus shown on MSCTA may be associated with RAAA. The main findings of the study were as follows: (1) the average CT value and the highest CT value of thrombus at the plane of MTD were higher in RAAA than in IAAA; (2) the thrombus volume in the range of 30 HU~150 HU was higher in RAAA than in IAAA, whereas the thrombus volume in the range of (−100 HU~30 HU) was not significantly different from that in IAAA; (3) The standardized thrombus volume in RAAA was positively correlated with thrombus in the range of 30 HU~150 HU, but not with thrombus volume in the range of −100~30 HU.

AAA is a disease caused by chronic inflammatory degenerative lesions arising in the aortic wall, characterized by progressive aneurysmal dilatation of the infrarenal aorta (20). The AAA with large diameters are often associated with thrombus (21), which can eliminate the endothelium and intima, progressive degeneration of the middle layer and inflammation or fibrosis of the outer layer (22). But the effect of ILT on the risk of AAA rupture is still controversial. Some studies suggest that ILT reduces aortic wall shear stress, thus acting as a mechanical cushion (10, 23, 24), being negatively correlated with the rate of AAA growth (25). Nevertheless, ILT may also create an inflammatory environment in which neutrophils, cytokines, proteases and reactive oxygen species are sequestered (26), which may cause a decrease in wall strength. Some clinical studies have even found that ILT correlates with the rate of swelling and rupture of AAA (27–29). Therefore, the weakening effect of ILT on the aneurysmal wall of AAA may be greater. However, although some studies have shown a possible correlation between ILT and RAAA, it has not been reported whether thrombus in different CT value ranges has a different effect on larger diameter in RAAA.

CT values of various plaque components in previous studies have been defined as low HU for vulnerable plaques, intermediate HU for fibrofatty plaques and high HU for fibrous or calcified plaques (30). Banno et al. (18) measured intraluminal thrombus density by using preoperative CTA in patients in a thoracic aortic aneurysm study and showed that low density thrombus/plaque may be one of the mechanisms causing the development of spinal ischemia after thoracic aortic stenting. It has also been shown in clinical computed tomography studies that the higher the calcium score, the higher the likelihood of future acute coronary events in patients (31). This shows that different densities may cause different diseases. Recent studies have also found that inflammation can contribute to thrombus formation (32), which in turn contains high levels of inflammatory factors (26), and that the thrombus is thus progressively thicker and denser, such that the high-density thrombus may reflect the degree of inflammatory weakening of the aneurysmal wall in patients. We observed the wall of the resected AAA in detail in the clinic and found that the wall of RAAA had a distinctive three-layer color, and the color of the wall of the aneurysm gradually deepened, which may be the result of different pathological responses to different densities of thrombus. Therefore, we speculate that the different densities of thrombus shown on MSCTA within the AAA may also have different effects on the RAAA as well.

In our study, firstly, we measured the CT values of thrombus at the plane of MTD in AAA and found that the average CT values and maximum CT values of thrombus in the RAAA group were significantly higher than those in the IAAA group (*P* < 0.05), and the maximum CT values in the RAAA group were still higher than those in the IAAA group in different diameter ranges of the tumor (*P* < 0.05). This can be seen that the high-density thrombus shown by MSCTA in RAAA may be an important factor causing rupture. Our findings are similar to some studies. For example, some studies have found that local bleeding from ILT predicts AAA rupture (33, 34), which may be due to the imaging findings of contrast leakage after intra-thrombotic hemorrhage, or intra-thrombotic hemorrhage has a high level of attenuation (34). To verify its diagnostic performance, we further did the ROC curve of RAAA and found that the AUC of the maximum CT value at the plane of MTD was 0.848, while the AUC value of the combined MTD with the maximum CT value at its plane predicted RAAA up to 0.901, which was a good prediction. Most of the AAA rupture risk assessments in previous studies were based on the maximum diameter of the abdominal aorta (4, 5), ignoring the effect of intraluminal thrombus on it. Although in recent years studies have begun to show that thrombus negatively affects AAA rupture (29), our study found that its influence is mainly caused by high-density thrombus rather than low-density thrombus.

As AAA is a specific larger volume of sac lumen, this study also innovatively calculated the respective volumes of ILT in AAA according to different ranges of CT values for the first time, and compared their similarities and differences in IAAA and RAAA. Overall, the MTD and total volume in the lumen of the abdominal aorta were higher in the RAAA group than in the IAAA, suggesting that larger diameter aortic aneurysms were still a risk factor for rupture in our clinic, in agreement with previous findings (35).

To more precisely understand the effect of ILT on RAAA, our study also calculated the thrombus volume within the lumen of the AAA (subrenal to bilateral common iliac artery bifurcation) at the range of different CT values and found that the total thrombus volume in the RAAA group was higher than that in the IAAA group. And the thrombus volume in the range of 30 HU~150 HU shown on MSCTA was higher in the RAAA group than in the IAAA group, while the thrombus volume in the range of −100 HU~30 HU was not significantly different between the two groups, which may indicate that the main growth in RAAA is still dominated by high-density thrombus and interacts with the transverse diameter in the aneurysm. Although we did note that there was no statistical difference between the two groups in the thrombus/plaque in the >150 HU range shown by MSCTA, the comparison between the two groups was not significant because the volume was so small and may have been outlined to encompass the plaque or AAA wall and not only the ILT. In previous studies, low-density thrombus shown on MSCTA in patients with thoracic aortic aneurysms may have been responsible for distal intercostal artery embolism (18). This may be due to the fact that low-density thrombus is finer and more likely to dislodge into the distal artery, whereas our study suggests that high-density thrombus shown on MSCTA may be thicker and more conductive, which in turn contributes to AAA rupture. In this study, we calculated the standardized thrombus as to rule out the confounding factors brought by the total volume in the AAA lumen. The results showed that the thrombus volumes in the range of 30 HU~150 HU and −100 HU~30 HU were both positively correlated with the standardized thrombus volume in IAAA, whereas only the thrombus in the range of 30 HU~150 HU was positively correlated with the standardized thrombus in RAAA. This suggests that the growth of thrombus in RAAA is dominated by high-density thrombus rather than low-density thrombus. In summary, the MTD combined with the maximum CT value at its plane predicts the occurrence of RAAA, and such a high-density thrombus may have a weakening effect on the aneurysm wall of AAA. Several mechanisms may explain this phenomenon here: (1) In contrast to low-density thrombus, whose sparseness is easily dislodged, MSCTA shows thicker high-density thrombus with higher shear rate (36) and stronger physiological force transduction (36–38); (2) High-density thrombus shown on MSCTA may be more isolated from neutrophils, cytokines, proteases, and reactive oxygen species than low-density thrombus (26); and (3) Increased high-density thrombus shown on MSCTA may accelerate apoptosis of active smooth muscle cells in the aortic wall and enhance local tissue hypoxia (39).

To our knowledge, this is the first report to address the association between thrombus in the range of different CT value and RAAA. Although the association between thrombus and RAAA has been clearly established (35, 40), our study further found that the MTD combined with the maximum CT value of thrombus at its plane predicted the occurrence of RAAA and high-density thrombus shown on MSCTA was closely associated with RAAA rather than low-density thrombus shown on MSCTA, which may provide a clinical rationale for delaying the progression of AAA or predicting precursor rupture of AAA in the future. In addition, compared with the previous reliance on CTA for imaging observation alone, this study provides an idea to study the potential association with RAAA by quantifying the combined MTD with thrombus CT values through MSCTA, which has the advantages of being objective, economic and reliable. Finally, there is a controversy in the scientific community regarding anti-thrombus therapy for AAA (41), and in the study it was shown that high-density thrombus shown on MSCTA may be associated with RAAA. Anti-thrombus therapy will probably prevent the appearance of high-density thrombus or its further growth in volume and thus reduce the risk of rupture of AAA, which needs to be confirmed in future prospective studies with large samples.

There are several shortcomings that need to be carefully considered. Firstly, this is a retrospective single-center cross-sectional study without a sufficiently large sample, and prospective studies are needed to justify this; Secondly, a majority of patients with RAAA die before arriving at the hospital, so there is a possibility of selection bias; Thirdly, as almost all of the RAAA admitted to our center had aneurysm diameters >60 mm, there is a need for further research as to whether the different thrombus densities of small aneurysms (<55 mm) likewise negatively affect AAA rupture; Fourthly, as there was only 1 case of non-ILT in the RAAA at our hospital, we excluded all cases of non-ILT to prevent bias. Because of this, our study was only applied to AAA patients with ILT. Finally, although we believe that the high-density ILT shown on MSCTA is a factor associated with weakening of the aortic wall, we have no tissue studies in this study to demonstrate this.

## Conclusion

High-density ILT shown on MSCTA in larger diameter AAA is associated with aneurysm rupture, and its MTD combined with the maximum CT value in its plane are a better predictor of RAAA. High-density ILT may be a major factor causing aortic wall degeneration and a characteristic of high-risk aneurysms, and anti-thrombus treatment of AAA associated with ILT may reduce the formation of high-density thrombosis, especially to provide a clinical basis for the current controversy in the scientific community regarding anti-thrombus treatment of AAAs.

## Data availability statement

The original contributions presented in the study are included in the article/supplementary material, further inquiries can be directed to the corresponding authors.

## Ethics statement

The study involving human participants was reviewed and approved by the Ethics Committee of Drum Tower Hospital (2022-556-01). Written informed consent from patients was not required for this retrospective study because the identifying information of patients was confidential.

## Author contributions

HL and ZC were involved in data preparation, drafting of the original manuscript, and designed the study. HL performed the statistical analysis. CT is responsible for the data supplementation. JC was responsible for the clinical evaluation. HF and XM were responsible for the MSCTA in which the regions of interest were outlined. TQ revised and confirmed the final article. All authors contributed to the article and approved the submitted version.

## Funding

This work was supported in part by the National Nature Science Foundation of China Grant (81870348).

## Conflict of interest

The authors declare that the research was conducted in the absence of any commercial or financial relationships that could be construed as a potential conflict of interest.

## Publisher's note

All claims expressed in this article are solely those of the authors and do not necessarily represent those of their affiliated organizations, or those of the publisher, the editors and the reviewers. Any product that may be evaluated in this article, or claim that may be made by its manufacturer, is not guaranteed or endorsed by the publisher.
